# Mechanisms of Coronavirus Cell Entry Mediated by the Viral Spike Protein

**DOI:** 10.3390/v4061011

**Published:** 2012-06-20

**Authors:** Sandrine Belouzard, Jean K. Millet, Beth N. Licitra, Gary R. Whittaker

**Affiliations:** 1 Center for Infection and Immunity of Lille, CNRS UMR8204, INSERM U1019, Institut Pasteur de Lille, Université Lille Nord de France, 59000 Lille, France; Email: sandrine.belouzard@ibl.fr; 2 Department of Microbiology and Immunology, Cornell University, Ithaca, NY 14853, USA; Email: jkm248@cornell.edu (J.K.M.); bnm4@cornell.edu (B.N.L.)

**Keywords:** coronavirus, spike, viral entry, fusion, proteolytic activation

## Abstract

Coronaviruses are enveloped positive-stranded RNA viruses that replicate in the cytoplasm. To deliver their nucleocapsid into the host cell, they rely on the fusion of their envelope with the host cell membrane. The spike glycoprotein (S) mediates virus entry and is a primary determinant of cell tropism and pathogenesis. It is classified as a class I fusion protein, and is responsible for binding to the receptor on the host cell as well as mediating the fusion of host and viral membranes—A process driven by major conformational changes of the S protein. This review discusses coronavirus entry mechanisms focusing on the different triggers used by coronaviruses to initiate the conformational change of the S protein: receptor binding, low pH exposure and proteolytic activation. We also highlight commonalities between coronavirus S proteins and other class I viral fusion proteins, as well as distinctive features that confer distinct tropism, pathogenicity and host interspecies transmission characteristics to coronaviruses.

## 1. Introduction

Although the first member of the coronavirus family was discovered in the 1930s [1] coronaviruses gained particular notoriety when the severe acute respiratory syndrome (SARS) outbreak shook the world in 2002–2003. Interest in this family of viruses grew in the aftermath of this epidemic, leading to the identification of many new family members. This episode also shed light on the capabilities of coronaviruses to jump across species. Before gaining importance for public health in 2003, the diseases associated with coronaviruses were mainly of veterinary interest. Coronaviruses infect a wide variety of mammals and birds, causing respiratory and enteric diseases and, in some rarer cases, hepatitis and neurologic disease. Infection can be acute or persistent [2].

Coronaviruses are classified in four different genera, historically based on serological analysis and now on genetic studies: alpha-, beta-, gamma-, and delta-CoV (Table 1). Coronaviruses belong to the *Coronavirinae* subfamily that together with *Torovirinae* form the *Coronaviridae* family in the *Nidovirales* order.

**Figure 1:**
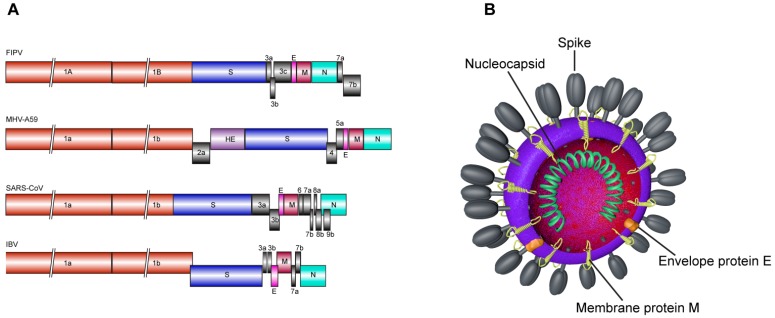
Coronavirus genomes (**A**). The genome of four different coronaviruses is depicted. The open reading frame (ORF)1a/b is colored in red. The HE gene present in MHV-A59 is represented in purple. The gene of structural proteins S (blue), E (pink), M (dark pink) and N (cyan) are localized in the 3' part of the genome. ORFs encoding accessory proteins are represented in grey. Coronavirus virion structure (**B**).

Coronaviruses are enveloped, spherical or pleiomorphic viruses, with typical sizes ranging from 80 to 120 nm. They possess a 5' capped, single-strand positive sense RNA genome, with a length between 26.2 and 31.7 kb, the longest amongst all RNA viruses. The genome is composed of six to ten open reading frames (ORFs). The first ORF comprises two-thirds of the genome and encodes the replicase proteins, whereas the last third contains the structural protein genes in a fixed order: (HE)-S-E-M-N (Figure 1A). Variable numbers of ORF encoding accessory proteins are present between these genes. The genome is packaged into a helical nucleocapsid surrounded by a host-derived lipid bilayer. The virion envelope contains at least three viral proteins, the spike protein (S), the membrane protein (M) and the envelope protein (E) (Figure 1B). In addition, some coronaviruses also contain a hemagglutinin esterase (HE). Whereas the M and E proteins are involved in virus assembly, the spike protein is the leading mediator of viral entry. The spike protein is also the principal player in determining host range [3,4]. 

Viral entry relies on a fine interplay between the virion and the host cell. Infection is initiated by interaction of the viral particle with specific proteins on the cell surface. After initial binding of the receptor, enveloped viruses need to fuse their envelope with the host cell membrane to deliver their nucleocapsid to the target cell. The spike protein plays a dual role in entry by mediating receptor binding and membrane fusion. The fusion process involves large conformational changes of the spike protein. Coronaviruses use a variety of receptors and triggers to activate fusion, however fundamental aspects that enable this initial step of the viral life cycle are conserved. In this review, we will address entry strategies of coronaviruses and how these mechanisms are related to host tropism and pathogenicity.

**Table 1:** Coronavirus genera, species and host receptor usage.

Genus	Species		Receptor
**Alphacoronavirus**	• Alphacoronavirus 1 comprising:		
		Feline Coronavirus (FCoV) serotype 2	Aminopeptidase N
		Canine Coronavirus (CCoV) serotype 2	Aminopeptidase N
		Transmissible gastroenteritis virus (TGEV)	Aminopeptidase N
	• Human coronavirus 229E		Aminopeptidase N
	• Human coronavirus NL63		ACE2
	• Porcine Epidemic Diarrhea Coronavirus (PEDV)		Aminopeptidase N
	• Rhinolophus bat coronavirus HKU2		
	• Scotophilus bat coronavirus 512/05		
	• Miniopterus bat coronavirus 1		
	• Miniopterus bat coronavirus HKU8		
**Betacoronavirus**	• Betacoronavirus 1 comprising:		
		Bovine coronavirus (BCoV)	Neu 5,9 Ac2
		Human coronavirus OC43 (HCoV-OC43)	Neu 5,9 Ac2
		Equine coronavirus (ECoV)	
		Human enteric coronavirus (HECoV)	
		Porcine haemagglutinating encephalomyelitis virus (PHEV)	
		Canine respiratory coronavirus (CrCoV)	
	• Murine coronavirus comprising:		
		Existing species of mouse hepatitis virus (MHV)	CEACAM1
		Rat coronavirus	
		Puffinosis virus	
	• Human coronavirus HKU9		
	• Rousettus bat coronavirus HKU4		
	• Tylonycteris bat coronvirus HKU5		
	• SARSr-CoV (SARS related Coronavirus) comprising		
		Human SARS-CoV	ACE2
		Rhinolophus bat viruses	
**Gamma-coronavirus**	• Avian coronavirus comprising:		
		IBV Various coronaviruses infecting turkey, pheasant, duck, goose and pigeon	
	• Beluga Whale coronavirus SW1		
**Delta-coronavirus**	• Bulbul coronavirus HKU11		
	• Thrush coronavirus HKU12		
	• Munia coronavirus HKU13		

## 2. Spike Protein

**Figure 2:**
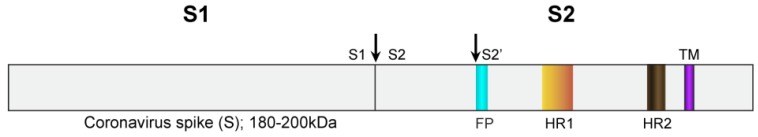
Severe acute respiratory syndrome (SARS)-CoV spike protein schematic. The spike protein ectodomain consists of the S1 and S2 domains. The S1 domain contains the receptor binding domain and is responsible for recognition and binding to the host cell receptor. The S2 domain, responsible for fusion, contains the putative fusion peptide (blue) and the heptad repeat HR1 (orange) and HR2 (brown). The transmembrane domain is represented in purple. Cleavage sites are indicated with arrows.

**Figure 3:**
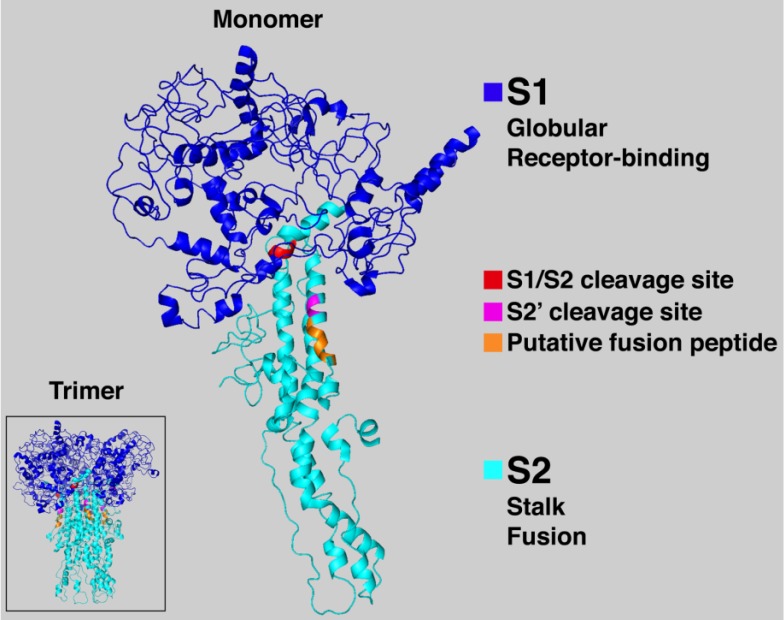
SARS-CoV spike protein three-dimensional predicted structure. This representation, shows the trimeric (inset) and monomeric forms of the protein and is based on the three-dimensional predicted model found in the PDB database (PDB entry 1T7G, [109]). Note that the predicted model does not include residues 681-736 of the protein. The S1 and S2 domains as well as the cleavage sites and putative fusion peptide are highlighted.

The spike protein is a large type I transmembrane protein ranging from 1,160 amino acids for avian infectious bronchitis virus (IBV) and up to 1,400 amino acids for feline coronavirus (FCoV). In addition, this protein is highly glycosylated as it contains 21 to 35 N-glycosylation sites. Spike proteins assemble into trimers on the virion surface to form the distinctive “corona”, or crown-like appearance. The ectodomain of all CoV spike proteins share the same organization in two domains: a N-terminal domain named S1 that is responsible for receptor binding and a C-terminal S2 domain responsible for fusion (Figures 2 and 3). A notable distinction between the spike proteins of different coronaviruses is whether it is cleaved or not during assembly and exocytosis of virions. With some exceptions, in most alphacoronaviruses and the betacoronavirus SARS-CoV, the virions harbor a spike protein that is uncleaved, whereas in some beta- and all gammacoronaviruses the protein is found cleaved between the S1 and S2 domains, typically by furin, a Golgi-resident host protease (Figure 2). Interestingly, within the betacoronavirus mouse hepatitis virus (MHV) species, different strains, such as MHV-2 and MHV-A59 display different cleavage requirements. This has important consequences on their fusogenicity, as detailed in Section 4. The S2 subunit is the most conserved region of the protein, whereas the S1 subunit diverges in sequence even among species of a single coronavirus. The S1 contains two subdomains, a N-terminal domain (NTD) and a C-terminal domain (CTD). Both are able to function as receptor binding domains (RBDs) and bind variety of proteins and sugars. 

The coronavirus spike protein is a class I fusion protein [5]. The formation of an α-helical coiled‑coil structure is characteristic of this class of fusion protein, which contain in their C-terminal part regions predicted to have an α-helical secondary structure and to form coiled-coils. Influenza hemagglutinin protein HA is the prototypical member of the class I fusion protein family and one of the best characterized so far [6]. HA is synthesized as a HA0 precursor and assembles into trimers. The protein becomes fusion competent by cleavage of HA0 into HA1 and HA2. The fusion peptide, a very conserved hydrophobic sequence, is located at the N-terminus of HA2. In the pre-fusion conformation, the central coiled-coil of the trimer is formed by three long helices with three shorter helices packed around them. In this conformation, the fusion peptide is protected, buried within the trimer interface. Two major conformation changes occur during fusion. Upon endosomal acidification, an unstructured linker becomes helical allowing formation of a long helix in the N-terminal part. In this conformation, called a prehairpin, the fusion peptide is projected towards the target membrane where it is then embedded, connecting the viral and target cell membranes. The second conformational change consists of the inversion of the C-helix that packs into the grooves of the N-terminal trimeric coiled-coils forming a six-helix bundle (6HB). In the resulting conformation, the transmembrane domain and the fusion peptide anchored into the target membrane are brought in close proximity facilitating merging of viral and cell membranes.

Coronavirus spike proteins contain two heptad repeats in their S2 domain, a feature typical of a class I viral fusion proteins. Heptad repeats comprise a repetitive heptapeptide ***a****bc**d**efg* with ***a*** and ***d*** being hydrophobic residues characteristic of the formation of coiled-coil that participate in the fusion process. For SARS-CoV and MHV, the post-fusion structures of the HR have been solved; they form the characteristic six-helix bundle [7,8]. The functional role of MHV and SARS-CoV HR was confirmed by mutating key residues and by inhibition experiments using HR2 peptides [9,10]. 

The important role of the spike protein in cell tropism has been demonstrated with chimeric viruses. There are many strains of mouse hepatitis virus (MHV), viruses that infect mainly the brain and liver. Because of the different patterns of disease associated with the various strains of MHV, involvement of their spike protein in tissue tropism has been extensively studied. The strain JHM is highly virulent causing severe encephalitis that is often lethal, but is poorly hepatotropic. The strain MHV-A59 causes hepatitis and mild encephalitis. MHV-2 is highly hepatotropic. By using chimeric viruses between these different strains, it has been shown that the S protein is linked to the tropism and pathogenesis of MHV. Introduction of JHM or MHV-2 S genes in the MHV-A59 background increases the recombinant virus’ neurovirulence and hepatotropism respectively [11,12]. However, replacement of JHM S protein sequence with MHV-A59 S gene in the JHM background does not confer hepatotropism suggesting that other factors modulate virus tropism. A mutant MHV-A59 strain exhibiting altered tropism was isolated from persistently infected microglial cells [13]. The single mutation Q159L in the S1 domain is responsible for reduced replication in the liver and low hepatotropism of the virus [14]. The important role of the spike protein in tropism has also been shown for other coronaviruses. IBV is an important domestic fowl pathogen that replicates in the respiratory tract but also in epithelial cells from the kidney, the oviduct and the gut. *In vitro*, clinical strains of IBV infect only chicken embryo kidney cells and grow on embryonated eggs. IBV Beaudette strain is an attenuated strain that was obtained by serial passage of IBV on eggs. IBV Beaudette, in addition to chicken embryo kidney cells, also infects CEF, BHK-21 and Vero cells. Substitution of the S gene in the Beaudette background with that of the IBV M41 strain restricts the tropism of the virus to primary chicken cells [15]. However, *in vivo* this chimeric virus has the attenuated phenotype of Beaudette. These data show that change in tropism of Beaudette in cell culture is mainly determined by the S protein though the avirulence also results from attenuating mutations in other genes [16]. 

Feline coronaviruses (FCoV) provide a fascinating example of the critical involvement of the spike protein in tropism and pathogenesis [17]. Within this alphacoronavirus species, there are two known serotypes, 1 and 2, based on serological and genetic characteristics of their spike. Furthermore, there are two biotypes within each serotype, both of which are associated with extremely contrasting pathological potential. Cats get commonly infected with the feline enteric coronavirus (FECV), a biotype that gives rise to usually asymptomatic to mild enteric tract infections and may establish persistence in the host. In contrast, some FCoV-infected cats sporadically develop an invariably fatal immune-mediated disease called feline infectious peritonitis (FIP). In this case, the causative agent is called feline infectious peritonitis virus (FIPV). A striking characteristic of FIPVs that sets them apart from FECVs is their ability to efficiently replicate in monocytes and macrophages [18]. It is thought that this switch in tropism, from gut epithelium to motile monocytes/macrophages cells, is a crucial tipping point towards the development of FIP as it allows for viral dissemination throughout the host.

The current understanding is that mutations in FECV in a persistently infected host cause it to change into the virulent FIPV [19]. While it has been hypothesized that mutations or deletions in certain genes, such as 3c and 7b, may be associated with the emergence of FIPV [19], the causative mutations for the biotype switch are still unknown. There is, however, evidence that mutations in the spike gene may play key role in the transition of tropism from gut epithelium to macrophages. Rottier and colleagues have focused on the genetically close and laboratory-adapted type 2 FECV 79–1683 and FIPV 79–1146 pair [20]. While both viruses have similar growth characteristics in established feline epithelial cells, only FIPV 79–1146 but not FECV 79–1683 has the ability to efficiently infect and replicate in macrophages. Using a targeted RNA recombination system [21] the authors were able to generate recombinant chimeric virus to determine regions of the genome that are important for infection of bone marrow derived macrophages. They found that the exchange of the FIPV 79–1146 S gene with that of FECV 79–1683 in the FIPV 79–1146 genetic background strongly reduced the chimeric recombinant virus’ capacity to infect macrophages compared to the recombinant wild type FIPV 79–1146. Furthermore, additional chimeras were generated to more precisely map the regions of spike that are important for macrophage tropism. Surprisingly, the C-terminal region of the spike (from residue 874 to the C-terminus) but not the N-terminal region (which contains the S1 receptor binding domain) was found to be responsible for the macrophage tropism in this system. A total of ten amino acid substitution differentiates the C-terminal regions of FECV 79–1683 and FIPV 79–1146, however the precise mutation(s) that cause the tropism switch remain(s) to be determined [20].

While serotype 2 FCoV have been studied in a relatively detailed manner, in particular because they propagate more easily *in vitro*, serotype 1 FCoV, which are more relevant clinically as they are more prevalent, are less well understood. And although it can be assumed that viruses of both serotypes behave in similar ways for most of their life cycle, it remains to be investigated whether the same or different set of mutations would account for the biotype switch in the two serotypes. Thus, more efforts are needed to study serotype 1 FCoV. Such efforts would shed light on the basis of FCoV pathogenesis.

The difference in tropism mediated by S proteins results from different mechanisms linked to the two main functions of the protein: receptor binding and fusion, which will be further discussed.

## 3. Receptor Binding and Tropism

The first coronavirus receptor identified was the MHV receptor, in 1991 [22]. MHV binds to the adhesion molecule CEACAM1 (Carcinoembryonic antigen-cell adhesion molecule) to infect cells. CEACAM1 is a type I transmembrane protein belonging to the immunoglobulin superfamily. CEACAM1 is a multifunctional protein that has roles in adhesion and cell signaling, among others. The CEACAM1 ectodomain contains four Ig constant region like domains, N, A1, B and A2. The N‑terminal domain N of CEACAM1 is involved in MHV binding [23,24]. There are two allelic forms of CEACAM1, CEACAM1a and CEACAM1b. They can both function as receptors, however, binding by CEACAM1a is much more efficient [23]. The involvement of receptor usage and tropism of MHV strains have been studied. It has been shown that neurovirulence of JHM is associated with rapid spread of the virus in the brain that is partly independent of CEACAM1. *In vitro*, MHV-JHM requires CEACAM1 for entry, however, *in vivo*, JHM is able to infect ceacam−/− mice but with a 100-fold higher lethal dose [25]. As a consequence, it has been suggested that JHM, in the absence of CEACAM1, uses an alternative, less effective and yet to be determined receptor in order to initiate infection. After primary infection, the virus could propagate very rapidly by using cell-cell fusion independently of the receptor (receptor independent spread) [26]. *In vivo*, MHV-A59 is strictly dependent on CEACAM1 for infection [27], but persistent infection of murine cells leads to the emergence of viruses with extended tropism [28]. The MHV/BHK virus infects cells in a heparan sulfate-dependent and CEACAM1-independent manner because of the acquisition of two heparan sulfate binding sites in the S protein [29,30]. It has been shown that both binding sites are required to acquire the CEACAM1-independent phenotype [30].

For JHM strain, many isolates exist that differ in their neurovirulence levels. The virulence is correlated to the length of a hypervariable region present within S1. The isolate MHV-4 of JHM contains the longest region and it is associated with independent CEACAM1 cell-cell fusion and spread [31]. It has been suggested that conformational changes of the spike protein are facilitated by a less stable association of the S1 and S2 subunits [32]. This suggests that the higher the fusogenic potential of the spike protein is, the less the virus depends on its receptor for entry. 

Among alphacoronaviruses two human viruses (HCoV-229E and HCoV-NL63) can be found along with viruses that infect animals and can be responsible of severe illness: transmissible gastroenteritis CoV (TGEV) and canine CoV (CCoV) cause enteric disease in pigs and dogs respectively while feline coronaviruses cause enteric and systemic disease in cats.

HCoV-229E, TGEV, serotype 2 FCoV and CCoV all use the aminopeptidase N (APN) protein of their natural host as receptor. Interestingly, in addition to their specific host APN, these viruses are able to bind the feline APN. It has been suggested that these viruses may have originated from a common ancestor coronavirus infecting felines that used APN as a receptor [2]. APN, also known as CD13, is a type II transmembrane protein expressed on the apical domain of epithelial cells of respiratory and enteric tracts. APN is a Zn^2+^ dependent protease that preferentially degrades peptides or proteins with a N-terminal neutral amino acid. It has been shown that tropism differences of these viruses are due to the ability of their spike proteins to recognize small species-specific amino acid differences in APN [33]. Spike proteins of HCoV-229E, TGEV, FCoV and CCoV present a high homology, however, binding domains are located in non-homologous regions. 

TGEV infects epithelial cells from the small intestine but is also able to infect cells from the respiratory tract. In the mid-1980s, an attenuated variant of TGEV, porcine respiratory coronavirus (PRCoV) was isolated in Belgium. This virus provides an example of altered tissue tropism due to a deletion occurring in the spike gene [34]. Unlike TGEV, PRCoV infects only pulmonary epithelial cells. Both spike proteins bind porcine APN, the receptor binding domain being located between residues 522 and 744 of TGEV S protein. The spike protein of TGEV has a hemagglutinating activity that is absent in PRCoV as this activity is contained in the deleted N-terminal part of the protein [35]. One of the consequences of this lack of activity is the inability of PRCoV to replicate in the gut. The hemagglutinating activity was mapped to the residues 145–155 of TGEV spike protein and it has been shown that a mutation abrogating this activity reduced the enteropathogenicity of the virus [36]. In addition, a study has shown that the sialic acid binding activity of TGEV is responsible for binding of an additional protein designated as mucin-like glycoprotein (MPG) in brush border membranes [37]. It has been suggested that this binding might shield the virus from the action of gut emulsifiers [38]. The role of the NTD and carbohydrate binding in TGEV provides interesting insights into coronavirus enterotropism, a property that generally is attributed to non-enveloped viruses. The role of the NTD in other enteric alphacoronaviruses such as FCoV and canine coronavirus (CCoV) is still unknown. 

Other coronaviruses have sialic acid binding activity, in particular bovine coronavirus (BCoV) and human HCoV-OC43 [39]. The ability of betacoronaviruses to bind carbohydrates has been mapped to a galectin fold-like structure present in the S1 NTD [40]. So far, besides the binding of Neu5,9Ac2 conjugates, no other specific receptors have been identified for these viruses. They belong to the betacoronavirus group and contain HE proteins, so they resemble influenza virus as they have a receptor-destroying enzyme. However, the exact role of HE during coronavirus entry remains unclear. IBV also exhibits sialic acid binding activity but the role of such activity in pathogenicity is not known. For IBV, extended host range of Beaudette strains in cell culture has been linked to the presence of a heparin binding site in the spike protein [41]. 

Another example of heparan sulfate binding is found with type 1 FCoV spike. By incubating viruses with heparin-agarose beads (heparin has a very similar structure to heparan sulfate, and is used in binding assays) de Haan and colleagues have demonstrated by quantification of bead-associated viral RNA that the cell-culture-adapted type 1 FIPV UCD1 strain can bind heparin [42]. Very interestingly, the putative heparin binding motif proposed by the authors resides in a defective furin cleavage site at the boundary between the S1 and S2 domains. The type 2 FIPV 79–1146 as well as the UCD1-related type 1 FIPV UCD, which harbors a functional furin cleavage site, were not able to bind the heparin beads. This lends support to the notion that an uncleaved heparan sulfate recognition motif is required for binding activity. Furthermore, the authors found that inoculation of UCD1 to FCWF cells in the presence of competing heparin severely diminished infection. Infection by type 2 FIPV 79–1146 was not affected by this heparin competition assay.

As mentioned above, the coronavirus spike protein/receptor pairing is a key determinant of tropism. To infect a new host species, coronaviruses must adapt to the receptor of their new host either by mutation or by recombination with a coronavirus infecting their new host. In the case of SARS-CoV, the virus appeared in 2002 in live animal retail markets in China. Related viruses were isolated from Himalayan palm civets, raccoon dogs and Chinese ferrets; however, it is believed that these animals were not the reservoir of the virus, but intermediate hosts during the species-jumping event. The receptor of the SARS-CoV is the angiotensin-converting enzyme 2 (ACE2) [43]. ACE2 is a type I integral membrane protein abundantly expressed in lung tissue; it is a mono-carboxypeptidase that hydrolyses angiotensin II. Human and Himalayan palm civet coronavirus receptor usage analyses have shown that human SARS-CoV can bind both human and palm civet ACE2 whereas the palm civet virus cannot bind hACE2. It has been shown that adaptation of the virus to humans was due to two point mutations, K479N and S487T, in the binding domain of the SARS-CoV S protein [44]. Further characterization by Wu *et al*. of adaptive mutations of the RBD led to the identification of mutations that strengthen the interaction with either human or palm civet ACE2 [45]. SARS-CoV-like viruses have been isolated in bats. In this case, entry does not occur via ACE2 and their receptor(s) is/are unknown; however, replacement of the amino acid sequence found between residues 323 and 505 with the corresponding sequence of the SARS-CoV RBD is sufficient to allow human ACE2 receptor usage [46].

Coronaviruses are able to exploit many cell surface molecules—proteins and carbohydrates alike—in order to gain entry into target cells. Host calcium dependent (C-type) lectins have been recognized to play a role in infection by SARS-CoV, IBV, and FCoV. Dendritic cell-specific intercellular adhesion molecule-3-grabbing non-integrin (DC-SIGN) is a C-type lectin expressed on macrophages and dendritic cells. Its function is to recognize high mannose glycosylation patterns commonly found on viral and bacterial pathogens. Viral exploitation of DC-SIGN is best documented in HIV-1, which attaches via N-glycosylated residues on the surface of the virus. HIV-1 uses DC-SIGN to subvert the host immune defenses by entering and initiating infection of dendritic cells or macrophages directly (*in-cis*), or by traveling with the cell to lymph nodes where the virus is transferred to T-cells at the immunological synapse (*in-trans*). Like HIV-1 gp120, the coronavirus spike is heavily glycosylated providing the virus with the opportunity to interact with host lectins such as DC/L-SIGN. L-SIGN, which is expressed on endothelial cells of the liver as well as in the lung, has been reported to be an alternate receptor for SARS-CoV and HCoV-229E [47,48]. *In-trans* transmission of SARS-CoV by dendritic cells to susceptible target cells has been documented. Although the dendritic cells studied were capable of transferring infectious virions via a synapse-like structure, *in-cis* infection was not observed [49]. Site directed mutagenesis has identified glycosylation at the asparagine residues 109, 118, 119, 158, 227, 589, and 699 as critical for L-SIGN/DC-SIGN mediated entry [50]. 

FIPV is an example of a coronavirus that targets immune cells—specifically monocytes and macrophages—to achieve systemic spread. Infection of non-permissive cell types was achieved through exogenous expression of DC-SIGN, demonstrating that both type 1 and type 2 FIPVs use DC‑SIGN as a co-receptor or as an alternative receptor to fAPN, respectively [51,52]. In the case of IBV, experiments have demonstrated that DC-SIGN and the closely related L-SIGN enhance infection of otherwise non-permissive cells, in a sialic acid-independent fashion [53]. The role of lectins in IBV infection *in vivo* is undetermined. 

## 4. Entry and Fusion

Enveloped virus entry can occur directly at the cell surface after binding to the receptor or after internalization via endocytosis with fusion taking place in the endosomal compartment. Fusion of viral membranes with host membranes is driven by large conformational changes of the spike protein. Over time, coronaviruses have modified their spike proteins, leading to the diversity of triggers used to activate their fusion. These conformational changes can be initiated by receptor binding but may need additional triggers such as pH acidification or proteolytic activation. The mechanisms of coronavirus entry are complex and differ between coronavirus species and strains. For example, depending on the MHV strain, fusion can occur directly at the cell surface after receptor binding or after endocytosis. JHM strain MHV-4 fuses at neutral pH, but the virus was also detected in endosomal vesicles [54]. It is likely that MHV-4 is capable of entering directly at the cell surface or through the endosomal pathway. The choice of entry mechanism may depend on the cell type. In both cases, fusion is solely triggered by receptor binding. Indeed, it has been shown that incubation of JHM spike protein with a soluble form of the receptor CEACAM1 induces modifications in S hydrophobicity and conformational change of the S2 region [55,56,57]. In addition, JHM is able to spread from DBT cells to BHK cells that do not express the MHV receptor. Incubation with soluble receptor increases this receptor-independent propagation [58]. The capacity of the spike protein to fuse at neutral pH relies on the properties of the fusion machinery. A variant of MHV-4 isolated from persistently infected cells requires low pH exposure for productive infection. The difference of pH requirement for fusion between the mutant and the wild type virus was attributed to three point mutations in the heptad repeat regions [59]. Concerning MHV-A59 entry mechanisms, contradictory results have been reported. Qui *et al.* reported that parental strain of MHV-A59 was insensitive to lysomotropic agent whereas the recombinant strain containing the MHV-2 spike protein relies on low pH for entry [60]. Indeed, MHV‑2 entry requires low-pH-activated endosomal proteases (cathepsin B and L) and, if a cleavage site is introduced in MHV-2 spike protein, virus entry no longer requires these proteases; these data are in favor of pH-independent fusion induced by receptor binding. Eifart *et al*. challenged this scenario. Combining different approaches of infection and microscopy, the authors have shown that MHV-A59 infection is sensitive to lysomotropic agent and that the virus is internalized allowing initiation of infection, suggesting that pH acidification is required to trigger viral fusion [61]. It is likely that receptor binding is a key determinant of MHV entry, however the requirement for an additional fusion trigger remains unclear. For MHV-2, the endocytosis mechanism was further characterized: the virus is internalized by a clathrin-dependent pathway that does not depend on the eps15 adaptor [62].

Endosomal pH acidification is a fusion trigger for many viruses such as influenza virus and vesicular stomatitis virus (VSV). For many years, it was believed that IBV fusion occurs at neutral pH as infected cells form large syncytia at neutral pH. However, it has been shown by Chu et al. that infection is blocked by lysomotropic agent and that IBV fusion process was activated by low pH [63]. The authors directly assessed the pH dependence of IBV fusion in fluorescence dequenching assays. They showed that fusion occurs at acidic pH, with a half-maximal fusion rate occurring at pH 5.5. In order to infect cells, IBV enters by endocytosis. Inhibitory drugs of the clathrin-mediated pathway such as chlorpromazine also abolished IBV infection. IBV virions harbor cleaved spike proteins, with the cleavage occurring between the S1 and S2 domains. The IBV Beaudette strain has the peculiar feature to contain a second furin cleavage site in the S2 domain of the spike. Infection and syncytia formation is inhibited by the presence of a furin inhibitor. Mutation or deletion of the S1–S2 cleavage site in Beaudette spike protein delays virus propagation but does not abolish syncytia formation. These mutants are still sensitive to furin inhibition. Mutants containing a minimal cleavage site XXXR690/S are infectious, however they are dependent on serine proteases for productive infection [64]. 

The relationship between the cleavability of the coronavirus spike protein and its fusogenicity has been a source of debate for researchers for many years. When viral fusion proteins are expressed at the cell surface, activation of viral fusion results in cell-cell fusion and formation of giant multinucleated cells named syncytia. It is generally believed that syncytia formation, which involves cell membranes fusing together, reflects the fusion process between viral and host cell membranes. However, it now appears that cell-cell and virus-cell fusion mechanisms may differ. Indeed, because of differences in factors such as membrane curvature and/or density of viral envelope glycoproteins, the processes involved in cell-cell and virus-cell fusion may considerably differ mechanistically. Furthermore, formation of syncytia in infected cells is not observed with all coronaviruses. Cleavage of the fusion protein is a common characteristic of class I viral fusion proteins. For influenza, the nature of the cleavage site of HA is of great importance in virus pathogenicity. The cleavage event required to prime the protein for fusion can occur in the secretion pathway by furin or during infection by host proteases of the respiratory tract. Influenza virus strains that are cleaved by host furin are highly pathogenic as they cause systemic infection [65]. For coronaviruses, the relationship between cleavage of the S protein and its cell-cell fusion capability has been well established. Cleaved proteins show a higher propensity for cell-cell fusion. Introduction of a mutation H716D in the spike protein of MHV-A59 strongly impaired the cleavage of the protein and delayed cell-cell fusion [66]. MHV-2 strain spike protein is not cleaved and mutation of the sequence of MHV-A59 cleavage site with the corresponding sequence of MHV-2 S protein delays cell-cell fusion. Introduction of a cleavage site in MHV-2 spike protein induces the formation of syncytia at neutral pH. *In vitro* spike protein cleavage plays an important role in fusogenicity, however the role in virus-fusion and pathogenicity is less clear. For example, MHV-A59 bearing the mutation H716D is very similar to the wild type virus in terms of pathogenicity [67]. MHV-A59 viral stock produced in presence of a furin inhibitor to block spike cleavage enters cells with kinetics similar to the wild type virus [68]. Moreover, a study by Hingley and collaborators has shown that the spike of virus purified from liver homogenates of MHV-A59-infected mice was not cleaved, further adding evidence that proteolytic processing of spike is not essential for entry and spread *in vivo* [69]. For coronaviruses that have a spike that is cleaved by furin, it is important to note that this protease belongs to a family of enzymes called proprotein convertases (PCs), which has nine members [70]. Along with furin, six other PCs proteases, PC1, PC2, PC4, PC5, PACE4 and PC7, share the same basic recognition motif: R/K-[X]_0,2,4,6_-R/K (where X is any amino acid) [71]. It remains to be determined whether other PCs that are related to furin can also recognize and cleave coronavirus spike proteins. 

For some coronaviruses that harbor a non-cleaved spike protein on their surface, such as MHV-2 and SARS-CoV, it has been shown that they rely on endosomal proteases for productive entry. Indeed, MHV-2 entry depends on host cell cathepsin L and B [60]. This dependence is abolished by the introduction of a furin cleavage site between the S1 and S2 domains. For SARS-CoV, the link between cleavage and fusion is more complex (Table 2). It has been shown that SARS-CoV infection is inhibited by lysomotropic agents because of the inhibition of the low-pH-actived protease cathepsin L [72]. In addition cell-cell and virus-cell fusion can be triggered by trypsin treatment [73]. This led to the hypothesis that SARS-CoV fusion was triggered by proteolytic processing of the spike protein. It has been shown that different proteases enhance SARS-CoV infection *in vitro*: trypsin, thermolysin and elastase [74]. Analysis of SARS-CoV spike protein processing by trypsin and elastase had shed light on SARS-CoV fusion [75,76]. It was shown that trypsin activates fusion by sequential cleavage of the spike protein at two discrete sites. The first cleavage event at the S1–S2 boundary (R667) probably facilitates the second cleavage event at the position R797 (S2’ region) that is responsible for fusion activation [75,77]. The second cleavage occurs directly at the N-terminal extremity of the fusion peptide. Cleavage at R667 is dispensable for fusion activation but enhances cell-cell or virus-cell fusion. Elastase mediates cleavage at the residue T795, not directly next to the fusion peptide. SARS‑CoV spike protein shows a certain degree of plasticity in the position of the cleavage site for priming of fusion. Conversely, when influenza HA is cleaved by *Pseudomonas* elastase the cleavage position is shifted by one amino acid, which leads to fusion incompetency [78]. SARS-CoV S residue 795 is probably less accessible for cleavage than residue 797 and the fusion induced by cleavage at position 797 is more efficient. The difference of fusion efficacy may also result from its location at the N-terminus of the fusion peptide. These data suggest that fusion is modulated by spatial regulation of the cleavage site. It has been shown that cathepsin L cleaves the SARS-CoV spike protein in the S1–S2 boundary region at residue T678, however, so far, cleavage in the S2’ region has yet to be conclusively demonstrated [79].

SARS-CoV is able to fuse directly at the cell surface in the presence of relevant exogenous protease. It is believed that this route of entry is 100- to 1000-fold more efficient than the endosomal pathway [74]. The availability of proteases in the extracellular milieu is a key factor of tropism. SARS‑CoV is a respiratory pathogen and it has been known for a long time that proteases from the respiratory tract such as members of the transmembrane protease/serine subfamily (TMPRSS), TMPRSS2 or HAT (TMPRSS11d) are able to cleave influenza HA [80]. Indeed, TMPRSS2 and HAT (or TMPRSS11d) are both able to induce SARS-CoV fusion [81,82,83,84]. Infection of target cells with SARS-CoV S-pseudotyped virions is less sensitive to cathepsin inhibitors when target cells express TMPRSS2 [83,84]. SARS-CoV S-pseudotyped virions produced in cells expressing TMPRSS2 still rely on endosomal cathepsin for entry, while they are less sensitive to neutralizing antibodies. This effect was attributed to the release of spike fragments in the supernatant that lure antibodies and may be of great importance for the spread of the virus [83]. It has been shown that processing of the spike protein by HAT and TMPRSS2 may differ: HAT cleaves the SARS-CoV S protein mainly at R667 whereas TMPRSS2 cleaves the protein at multiple sites, notably in a region near S2’, although the precise locations of the cleavage sites for this protease remain to be determined [81]. Expression of HAT in target cells does not confer NH_4_Cl or cathepsin inhibitor resistance to SARS-CoV S‑pseudotyped virion entry [84]. It is likely that spatial and temporal modulation of activation by proteases play important roles. Cleavage of incoming virions before their binding to the cell would probably abort infection by inactivating the virion. Interestingly, TMPRSS2 is associated with ACE2, the SARS-CoV receptor [81]. TMPRSS2 likely plays a key role in the initial infection and spread of the virus; however the importance of this protease on SARS-CoV infection results from a fine balance between two antagonist effects on infection: shedding of the receptor and fusion activation.

**Table 2:** Proteolytic processing of the SARS-CoV spike protein important for cell-cell fusion and/or virus entry. The table summarizes the different proteases involved in the cleavage of SARS-CoV spike protein and their roles in activating cell-cell fusion and/or virus entry.

Protease	Cleavage site	Cell-cell fusion	Virus Entry
**Cathepsin L** [72,79]	**S1/S2** HTVSLLRSTSQKSIVAYTMSL	-	+
**Elastase** [74,85]	**S2’** LPDPLKPTKRSFIEDLLFNKV	+	+
**HAT (TMPRSS11d)** [84]	**S1/S2** HTVSLLRSTSQKSIVAYTMSL	+	-
**Plasmin** [82]	**S1/S2** HTVSLLRSTSQKSIVAYTMSL **S2’** LPDPLKPTKRSFIEDLLFNKV	n.d.	+
**TMPRSS11a** [82]	**S1/S2** HTVSLLRSTSQKSIVAYTMSL **S2’** LPDPLKPTKRSFIEDLLFNKV	n.d.	+
**TMPRSS2** [81,83,84]	Multiple sites	+	+
**Trypsin** [7373,^75^]	**S1/S2** HTVSLLRSTSQKSIVAYTMSL **S2’** LPDPLKPTKRSFIEDLLFNKV	+	+

For FCoV, a well-studied case for the role of proteolytic activation of spike comes from research on the type 2 FCoV pair FECV 79–1683 and FIPV 79–1146 [86]. By using specific cathepsin inhibitors, the authors have shown that the two strains differ substantially in their use of activating proteases used during entry. While the FECV strain 79–1683 was found to rely on both cathepsin B and L as well as on an acidic endosomal environment, the FIPV strain 79–1146 was dependent on cathepsin B activity only. This was further confirmed by a biochemical assay that found that FECV 79–1683 can be cleaved by cathepsin B and L, whereas FIPV 79–1146 could only be cleaved by cathepsin B. Based on the molecular weights of the cathepsin cleavage products, it was hypothesized that the cleavage site did not reside in the boundary region between the S1 and S2 domain, but in a region located in the C‑terminal part of S2 [86].

## 5. Fusion Peptide

A critical feature of any viral fusion protein is the so-called “fusion peptide”, which is a relatively apolar region of 15–25 amino acids that interacts with membranes and plays an essential role in the fusion reaction [6,87,88]. Fusion peptides of class I viral fusion proteins are typically classified as “external” or “internal” depending on their location relative to the proteolytic cleavage site [89]. One key feature of viral fusion peptides is that within a particular virus family, there is high conservation of amino acid residues; however, there is little similarity between fusion peptides of different virus families [90]. In the case of influenza HA, which is a classic example of an “external” fusion peptide, the N- and C-terminal parts of the fusion peptide (which are α-helical) penetrate the outer leaflet of the target membrane, with a kink at the phospholipid surface. The inside of the kink contains hydrophobic amino acids, with charged residues on the outer face [91]. Internal fusion peptides (such as the one found in the Ebola virus GP) often consist of loops, but also require a mixture of hydrophobic and flexible residues similar to N-terminal fusion peptides [87,92]. It is important to note that despite the presence of key hydrophobic residues, viral fusion peptides often do not display extensive stretches of hydrophobicity, and can contain one or more charged residues [93]. 

To date, the exact location and sequence of the coronavirus fusion peptide is not known [94], however, by analogy with other class I viral fusion proteins, it is predicted to be in the S2 domain. The location of the fusion peptide has been most extensively studied for SARS-CoV. Three membranotropic regions in SARS-CoV S2 were originally suggested as potential fusion peptides [95,96]. Based on sequence analysis and a hydrophobicity analysis of the S protein using the Wimley-White (WW) interfacial hydrophobic interface scale, initial indications were that the SARS-CoV fusion peptide resided in the N-terminal part of HR1 [5,97], which is conserved across the *Coronaviridae*. Mutagenesis of this predicted fusion peptide inhibited fusion in syncytia assays of S-expressing cells [98]. This region of SARS-CoV has also been analyzed by other groups in biochemical assays [96,99,100] and was defined as the WW II region (residues 864–886)—although Sainz *et al.* [99] actually identified another, less conserved and less hydrophobic, region (WW I, residues 770–778) as being most important for fusion. Peptides corresponding to this region have also been studied in biochemical assays by other groups [101]. In addition, a third, aromatic region adjacent to the transmembrane domain (the membrane-proximal domain) has been shown to be important in SARS-CoV fusion [102,103,104,105]. This membrane-proximal domain likely acts in concert with a fusion peptide in the S2 ectodomain to mediate final bilayer fusion once conformational changes have exposed the fusion peptide in the ectodomain.

Based on the finding that SARS-CoV S can be proteolytically cleaved at a downstream position in S2, at residue 797 [75,76,77], further investigations were carried out to determine whether cleavage at this internal position in S2 might expose a domain with properties of a viral fusion peptide. A mutagenesis study of SARS-CoV S residues 798–815, a stretch located in between the WW I and WW II regions, combined with lipid-mixing and structural studies of an isolated peptide, showed the importance of this region as a novel fusion peptide for SARS-CoV. The sequence immediately C‑terminal to the R797 cleavage site of SARS-CoV S is SFIEDLLFNKVTLADAGF, and it is notable that both R797 and this downstream sequence are highly conserved across the *Coronaviridae*. In particular, the IEDLLF motif showed only minimal divergence, with occasional conservative substitutions [106].

Examination of the proposed IEDLLF fusion peptide in the context of a structural model of the SARS-CoV S homotrimer shows that it is externally positioned mid-way down the trimer, and as such, would appear to be appropriately located to function as a fusion peptide. Notably, L803, L804 and F805 are the initial residues of a major antigenic determinant of SARS-CoV S (Leu 803–Ala 828) that is capable of inducing neutralizing antibodies [107]. This SARS-CoV epitope is also homologous to an immunodominant neutralizing domain (the 5B19 epitope) on the MHV S2 subunit [108]. While a crystal structure of the SARS-CoV S ectodomain has not yet be solved, a predictive model of the quaternary structure is available: PDB entry 1T7G [109]. In the context of this model, the novel S2 fusion peptide is mainly helical (especially the conserved residues SxIEDLLF), with a short central unstructured region, and is in a relatively exposed position mid-way down the trimeric spike protein complex (Figure 3). This structure and position within the S trimer is consistent with its function as a viral fusion peptide. Comparisons with other fusion proteins reveal some similarities to the “internal” fusion peptides of Ebola virus and avian leukosis virus. Like these viruses, the coronavirus fusion peptide is exposed by proteolytic cleavage [6,89,110], yet is not a classic “external” fusion peptide like influenza HA.

## 6. Conclusions

In the past ten years, many new coronaviruses have been identified. They infect a wide range of hosts from mammals to birds. Closely related coronaviruses have been identified in distantly related animals suggesting recent interspecies jumps. Coronavirus diversity is fundamentally due to the low fidelity of the virally encoded RNA-dependent-RNA-polymerase that generates around 10^−3^ to 10^−5^ substitutions per site per year. The large size and replication strategy of the coronavirus genome also allows for frequent homologous recombination, a process that enables exchange of genetic material during co-infection. Persistent infection leads to the accumulation of adaptive mutations. The consequences of coronavirus species barrier jumping can be devastating and result in severe disease and mortality, as exemplified by the SARS outbreak. The spike protein is the major determinant of coronaviruses tropism. Modification of the spike can alter cell and tissue tropism and, in some cases, in association with other viral and host factors, may lead to change of virus pathogenicity. Zoonoses constitute a real risk for human health. In the past, coronaviruses have often demonstrated their propensity to infect new hosts, highlighting the capacity for viral evolution and the need for surveillance. Great progress has been made in the understanding of spike protein functions, however it remains impossible to predict the effect of mutations that a virus might acquire.
